# Aberrant chromatin organization at the nexus of laminopathy disease pathways

**DOI:** 10.1080/19491034.2022.2153564

**Published:** 2022-12-11

**Authors:** Garrett T. Santini, Parisha P. Shah, Ashley Karnay, Rajan Jain

**Affiliations:** Departments of Medicine and Cell and Developmental Biology, Penn Cardiovascular Institute, Penn Epigenetics Institute, Perelman School of Medicine, University of Pennsylvania, Philadelphia, USA

## Introduction: the nuclear lamina is central to chromatin organization

The nucleus is a highly organized membrane-bound organelle that envelops and regulates multiple aspects of the genome. The nuclear membrane is composed of a complex lipid bilayer which features inner and outer surfaces, transmembrane proteins, and nuclear pores. The inner nuclear membrane surface interfaces with the nuclear lamina, an interlinked protein lattice structure that is integral to nuclear structural integrity and acts as a scaffold for chromatin organization [1,2]. Mutations in genes encoding the primary lamina component – nuclear lamin proteins – result in a range of syndromes collectively referred to as laminopathies. This class of diseases is characterized by a variety of clinical presentations, including myopathy, lipodystrophy, neuropathy, and segmental progeroid syndromes [3]. The molecular basis for clinical pathologies remains unclear, and current treatment regimens focus on ameliorating specific disease manifestations. Multiple mechanisms have been implicated in laminopathy phenotypes, including deregulated chromatin organization, compromised signal transduction, and aberrant mechanical transduction (hereafter mechanotransduction). The individual or combined contribution of these abnormalities to disease manifestation remains to be unraveled, but recent evidence has linked compromised genome architecture to disease in specific tissues. Advances in microscopy, genomics, and induced pluripotent stem cell (iPSC) model systems have enabled investigation of aberrant lamin-associated molecular pathways implicated in laminopathies and their contribution to disease. Here, we review the clinical phenotypes of various laminopathies and explore the epigenetic and chromatin-related consequences of *LMNA* disease-linked mutations.

## Clinical consequences: laminopathy phenotypes span multiple tissues

A number of laminopathy syndromes have been clinically defined, including dilated cardiomyopathy (DCM) [4], autosomal dominant Emery-Dreifuss muscular dystrophy (EDMD) [5], limb girdle muscular dystrophy (LGMD) [6], Charcot-Marie-Tooth disorder type 2b [7], and Hutchinson-Gilford Progeria Syndrome (HGPS) [8]. In particular, DCM has a high morbidity rate, often progressing to heart failure and sudden cardiac death [4]. The genetic mechanism by which pathogenic variants result in clinical phenotypes is varied. A majority of laminopathies are associated with heterozygous *LMNA* mutations, which include mostly missense and a smaller fraction of nonsense mutations that span the gene [9,10]. An exception to the myriad *LMNA* mutations is in HGPS patients, who overwhelmingly carry specific, truncating mutations in *LMNA* exon 11 [10]. Many pathogenic *LMNA* variants are thought to result in functional haploinsufficiency of lamin A and lamin C proteins (hereafter lamin A/C), encoded by *LMNA* [9,10]. This paradigm is partially based on a *Lmna^±^* murine model in which *LMNA* haploinsufficient mice develop DCM and conduction system disease [11,12]. Surprisingly, there is little discernible correlation between the location of mutation and the corresponding clinical phenotype for several laminopathy syndromes (with the exception of HGPS). Indeed, cardiac phenotypes have been attributed to mutations spanning exons 1–10 [13,14]. Adding complexity, some pathogenic variants manifest differently in patients; pathogenic *LMNA* T959 has resulted in DCM, EDMD-like skeletal muscular abnormality, and LGMD-like muscular dystrophy all within the same family [15]. Additional details regarding the clinical syndromes and genetics of laminopathies are well characterized in other reviews [3,10,16,17].

*LMNA* is expressed nearly ubiquitously across cell types, but many germline *LMNA* mutations disproportionately manifest in cardiac, muscular, adipose, and neuronal pathologies. A notable exception is HGPS, a pleiotropic disease associated with accelerated aging that affects multiple tissues and is overwhelmingly associated with a specific genetic mutation. It is possible that certain cell types are more vulnerable to *LMNA* aberrations because specific molecular pathways affected by *LMNA* mutation are more critical to these cells. Gaining understanding of the molecular pathogenesis of laminopathies may improve interventions for treatment. For example, laminopathy-associated DCM is currently treated at symptom onset, but it is plausible that treatment prior to symptom manifestation would prevent or delay the progression of heart failure and the need for transplant, improving the patient outcome and reducing the medical burden.

While pathogenic *LMNA* variations underpinning laminopathies have been clinically defined, it remains unclear how these mutations alter lamin A/C function and how the subsequent dysregulation impacts phenotypic outcomes. To date, there is strong evidence implicating genome organization, signal transduction, and mechanotransduction abnormalities in laminopathies [18,19]. Based on existing studies, there is room for significant debate over what drives pathology among these three general pathways. Lamin A/C is involved in a variety of signaling pathways [20,21], supporting a model in which laminopathies are diseases of signal transduction. In support of this, a foundational study in an EDMD murine model linked upregulated mitogen-activated protein kinase (MAPK) signaling to cardiomyopathy [21]. More recently, it has been demonstrated that MAPK inhibition delays the progression of *Lmna*-associated cardiomyopathy in this same murine model [22,23]. The contribution of signaling abnormalities in laminopathies is beyond the scope of this review and has been reviewed elsewhere [20,24,25]. It remains unclear what upstream phenomena lead to increased MAPK signaling, which molecular targets of MAPK signaling are impacted in laminopathies, whether specific proteins in the pathway are the most appropriate therapeutic target, and what the ideal range of time is in which an agent impacting signaling would be most effective. Recent Phase III trials with oral p38 inhibitors were terminated early, secondary to futility [26], but additional molecular details may prove to be helpful in shaping new interventions involving modulating MAPK signaling. Similarly, reductions or defects in lamin A/C are linked to a weakened nuclear membrane [27,28], which itself is a critical factor in mechanosensing [29], leading to models in which mechanical force underlies laminopathy progression [19]. A number of studies have explored the impact of mechanical stress on cells and are reviewed elsewhere [19,30,31], but within the scope of this review, it is interesting to consider that the epigenetic and chromatin architecture abnormalities observed in laminopathies, reviewed below, are common to multiple laminopathy disease pathways.

## Epigenetic consequences: pathogenic *LMNA* variants dysregulate genome organization

The nuclear lamina plays an important role in epigenetic gene regulation and genome organization. As such, it is unsurprising that pathogenic or haploinsufficient *LMNA* mutations have adverse effects on the epigenome [32]. Interestingly, though, changes occur across an epigenetic range, including three-dimensional (3D) organization, balance of post-translational histone modifications, and DNA methylation, reviewed below. The eukaryotic genome is highly organized, and proper organization is necessary for differentiation and development. Highlighting its importance, disruptions in nuclear architecture can contribute to disease [33–36]. Our understanding of the importance of 3D genome structure has significantly improved due to advances in mapping interacting chromosomal regions, including Hi-C [37–39]. A detailed review of 3D organization methods is beyond the scope of this review, but in brief, Hi-C is an evolution of Chromosome Conformation Capture (3C) technology. DNA is cross-linked to covalently link genomic regions that are proximal to each other in 3D space, then digested with unique restriction enzymes and deeply sequenced in order to produce contact matrices indicating non-adjacent areas of the genome which preferentially interact with one another [40]. Hi-C maps support an organizational model in which chromosomes are the largest unit of genome organization, occupying discrete territories within the nucleus. Organization can be further divided into compartments that are generally transcriptionally active (A compartment) and predominantly transcriptionally repressed (B compartment). Hi-C maps have also revealed areas that preferentially self-interact, known as topologically associating domains (TADs); within TADs are loops, the smallest unit of organization, in which two non-adjacent genomic areas on the same chromosome interact [37–39,41,42]. Several studies have implemented Hi-C to understand the impact of lamin mutations on 3D architecture. In murine embryonic stem cells (mESC) harboring deletions of *Lmna, Lmnb1*, and *Lmnb2*, TADs from triple knockout cells were highly similar to control, but the researchers observed significant changes in the interactions between TADs in the mutant compared to control cells [43]. Re-expressing lamin B1 in the triple knockout cells rescued the TAD-TAD interaction changes in the mutant cells, indicating a role for lamina proteins in normal genome architecture. Similarly, TAD-TAD interaction changes were also observed in a human iPSC-derived cardiac myocyte (hiPSC-cardiac myocyte) model of *LMNA* haploinsufficiency. The authors noted that reduction in lamin A/C reinforces separation between chromosome territories and chromatin compartments, with compartment changes restricted to approximately 1.2% of the genome [44].

The limited changes in 3D architecture in both the murine knockout and human haploinsufficient lamin mutants have challenged the hypothesis that chromatin disorganization is central to laminopathy pathogenesis [45], but there are limitations to generalizing these data with respect to disease. First, neither the mESC nor hiPSC-cardiac myocyte models feature a pathogenic missense mutant. Thus, any dominant or pathogenic variant-specific effect is not captured. Second, population-based Hi-C represents an average of interaction data cross a population, and any cell-to-cell heterogeneity and/or range of differences across cells are obscured [46]. Super-resolution microscopy has demonstrated remarkable genome organization heterogeneity under homeostatic and experimental conditions [47–49]. Third, it is challenging to determine what ‘dose’ of epigenetic disruption or insult is necessary to cause disease. For example, the *LMNA* haploinsufficient hiPSC-cardiac myocytes had inappropriate compartment changes (B to A) and expression of non-lineage genes, including *CACNA1A*, a calcium channel not normally highly expressed in the heart. Pharmacological inhibition of this channel improved the electrophysiologic abnormalities observed in the *LMNA* mutant cardiac myocytes [44], consistent with the hypothesis that compartment switches have functional consequences. Thus, it is possible that early dysregulation in compartment or TAD organization is exacerbated over time, contributing to phenotypes that often present several years after birth. Finally, while hiPSC models have proven to be extremely insightful in modeling disease, the derived cell types often represent immature states [50] and strategies to examine mature cells in a 3D context will undoubtedly advance our efforts.

*LMNA* mutations can also affect the epigenome independent of any impact on overall 3D organization. Lamin A interacts with a number of factors and complexes that mediate epigenetic regulation, including lamina-associated polypeptide 2α/β (LAP2α/β) and lamin B receptor (LBR), amongst others [2]. For the purpose of this review, we will detail one salient example representative of this type of epigenetic dysregulation which involves PRC2, a member of the Polycomb repressive complex. PRC2 catalyzes post-translational trimethylation of lysine 27 on histone H3 (H3K27me3) and mediates gene repression in a variety of developmental contexts, including adipogenesis and myogenesis [51,52]. Lamin A/C and PRC2 physically interact, and depletion of lamin A/C causes mis-localization of PRC2 in a murine myogenesis model [53]. This interaction was also shown to be affected in a hiPSC-cardiac myocyte model of a pathogenic *LMNA* K219T missense mutation [54] (hereafter K219T). Investigators utilized chromatin immunoprecipitation (ChIP) to map PRC2 and H3K27me3 in K219T and control cells. ChIP is a well-established method to map occupancy of DNA-crosslinked proteins of interest across the genome [55]. Salvarani and colleagues determined PRC2 and H3K27me3 co-occupy *SCN5A*, a critical sodium channel that is downregulated in K219T cells. The team employed a DNA fluorescence in situ hybridization (FISH) approach to identify the positioning of a probed region within the nucleus at single cell resolution [56]. Accordingly, the team found greater PRC2 occupancy at the *SCN5A* locus and a repositioning of the locus closer to the nuclear periphery using DNA FISH in K219T cells compared to wild type. The data suggested the K219T mutation results in some loci, including *SCN5A*, gaining PRC2 enrichment, causing inappropriate transcriptional repression [54]. Overall, these observations provide an example of how pathogenic *LMNA* mutations may perturb normal interactions with other proteins to result in epigenetic dysregulation.

*LMNA* mutations have consistently been linked to alterations in the levels of active and repressive post-translational histone modifications. Several studies have mapped enrichment of various histone modifications in lamin A/C mutant models including mESCs, hiPSC-cardiac myocytes, and adipocyte stem cells (ASCs) [43,54,57,58]. In the aforementioned K219T hiPSC-cardiac myocytes, decreased expression of *SCN5A* was associated with increased repressive modifications H3K27me3 and trimethylation of lysine 9 on histone H3 (H3K9me3) on the promoter of that gene [54]. Likewise, genes with increased expression in *LMNA* mutants show an associated enrichment in active histone modifications. In an hiPSC-cardiac myocyte *LMNA* haploinsufficiency model, there was increased deposition of the histone post-translational modification trimethylation of lysine 4 on histone H3 (H3K4me3), associated with gene activity, in the promoter of the upregulated gene *PDGFRB* [57]. Similarly, in an ASC model of *LMNA* R482W (hereafter R482W; lipodystrophy-causing missense mutation), the active post-translational modification acetylation of lysine 27 on histone H3 (H3K27ac) was enriched at the enhancer of *MIR335*, an aberrantly upregulated microRNA [59]. Another approach to studying histone modifications in *LMNA* mutants has been to examine the repressive dimethylation of lysine 9 on histone 3 (H3K9me2) modification, which is enriched at chromatin localized to the nuclear periphery [60,61]. Multiple *LMNA* missense and frameshift mutants result in the loss of H3K9me2 at the nuclear periphery by immunofluorescence (IF) and co-occupancy with lamin B1(LB1) [57,58]. Collectively, these studies indicate that gene expression changes in laminopathy cell models are associated with redistribution of histone modifications at loci hypothesized to contribute to pathogenesis, with notable evidence that peripheral heterochromatin is particularly disrupted.

In addition to changes in the distribution of histone modifications across the genome, lamin proteins themselves are subject to a variety of post-translational modifications. The full spectrum of lamin processing, possible modifications, and their functional consequences is beyond the scope of this review and is well reviewed elsewhere [62] One particularly compelling example is a recent study linking lamin phosphorylation to HGPS. In addition to localization at the nuclear periphery, a low concentration of lamin A/C is also normally present in the nucleoplasm [63]. It is well established that lamin A/C phosphorylation is involved in mitotic disassembly of the nuclear lamina, but phosphorylated lamin A/C is also found in the nucleoplasm in interphase [64,65]. A study in human fibroblasts revealed that lamin A/C phosphorylated on serine 22 (pLamin) binds to euchromatic, active enhancers. ChIP-sequencing approaches demonstrated H3K27ac enrichment at more than 80% of pLamin binding sites [66]. In fibroblasts derived from two HGPS patients, pLamin was enriched at enhancers associated with upregulated, pathogenic HGPS genes compared to age-matched control fibroblasts. These genes contribute to liver disease, arthritis, and coronary pathologies [66]. This and other examples support a model where one or more lamin A/C modifications contribute to HGPS pathogenesis, justifying deeper investigation into how such modifications are linked to laminopathies, particularly in pathogenic missense *LMNA* mutants that alter amino acid modification targets.

Finally, *LMNA* mutants have also been linked to epigenetic regulation defects at the level of DNA. Specifically, a study of patient-derived fibroblasts and paired hiPSC lines from two families with *LMNA* mutations identified thousands of differentially methylated genomic regions (DMRs) [67]. While a portion of these DMRs fell within normally silent areas of the genome, there was also significant enrichment for regulatory elements, including enhancers and promoters and multiple ‘epimutation’ hotspots in genes associated with lineages implicated in clinical laminopathies. Consistently, human myocardial studies showed changes in DNA methylation in DCM cardiac myocytes and mutant myocardium [68,69]. Collectively, these data and all the data reviewed in this section underscore the range of epigenetic changes in *LMNA* mutations. The highlighted studies and others establish evidence of epigenetic defects in pathogenic *LMNA* mutations, but it remains unclear if changes are uniform across different pathogenic mutations. Dedicated experiments using the same assays, cell types, and conditions to compare several different *LMNA* mutations are required to investigate the generalizability of these previous studies. More importantly, what is the relationship between the broad epigenetic dysregulation and disease onset? It remains an area of active research to understand how these various changes contribute to disease pathology. Doing so may inform our ability to target treatments that reverse or prevent pathogenic epigenetic changes in laminopathies.

## LAD dysregulation: lamina-associated chromatin is altered in laminopathy-modeled cells

As alluded to above, chromatin is radially organized, with some regions positioned at the nuclear periphery versus other regions localized more centrally within the nucleus. A subset of peripherally positioned chromatin physically contacts the nuclear lamina, in regions termed lamina-associated domains (LADs). In the following section, we will briefly summarize the molecular characteristics of LADs, and then review studies examining LADs in laminopathies.

LADs occupy ~30–40% of the total genome and are highly gene depleted; genes that are localized within LADs are typically transcriptionally silenced and enriched for hallmarks of heterochromatin, including H3K9me2 and H3K9me3 modifications [70–72]. In accord, LADs predominantly map to B compartment chromatin, as defined by Hi-C [73]. LADs do not contain an obvious defining DNA sequence motif, and tethering proteins that directly attach chromatin to the lamina remain elusive in higher eukaryotes [74]. Interestingly, LAD borders are characterized by increased gene density, higher gene expression, and enrichment for the boundary and insulating CCCTC-binding factor (CTCF), suggesting that boundaries between LAD and non-LAD chromatin may be dynamically or actively maintained [71,75,76]. Together, these features suggest that LADs are likely important for both genome organization and nuclear integrity. In addition, LADs are implicated in regulating gene transcription. A subset of LADs has been shown to have cell type specificity [77–79] and is linked to cell type-specific gene expression changes. In differentiating cells, LADs are radially re-positioned such that genes relevant to the acquired cell type are ‘released’ from the periphery and genes relevant to pluripotency and alternative fates become peripherally ‘sequestered’ within LADs [60,77,80,81]. These changes in radial positioning are thought to confer the transcriptional activation and repression of target genes, respectively. In contrast, constitutive LADs – regions that are stably retained as LADs between certain cell types [82] – are enriched in A/T base pairs and long-interspersed nuclear elements (LINEs), raising the possibility that LADs may also function to silence potentially deleterious repetitive elements within the genome [82–84]. Comprehensive molecular characteristics of LADs have been reviewed in detail [85–87]. In sum, LADs are linked to both transcriptional regulation and 3D genome organization.

Changes in LADs and spatial positioning appear to contribute to laminopathy phenotypes. *LMNA* mutations have been shown to cause extensive changes in LAD organization, including both the loss of normal LADs and the formation of unique LADs [58]. These disruptions are associated with two major phenotypes: impaired differentiation and non-lineage gene expression [58,88]. One series of studies characterized the effect of pathogenic lipodystrophy-associated R482W on LADs [59,89,90]. First, scientists demonstrated that expressing pathogenic R482W in adipocyte progenitors inhibits normal adipocyte differentiation, leading to upregulated expression of non-lineage myogenic genes [59]. Interestingly, the aberrant gene upregulation was not observed in adipocyte progenitors with *LMNA* knock-down, raising the possibility of a specific pathogenic dominant-negative effect [59]. Subsequent ChIP-seq revealed few changes in lamin A-defined LADs between R482W and wild-type fibroblasts. However, genes in areas where LADs were lost showed increased expression, while genes in areas where LADs were gained showed decreased expression [90]. In a specific example, the *T/BRACHYURY* (*TBXT*) gene, which encodes a mesoderm inducer, was located within a LAD in control fibroblasts but not in R482W patient-derived fibroblasts [89]. The team then engineered hiPSC lines from an R482W patient and generated isogenic corrected lines to determine the impact of the mutation. In contrast to the K219T studies, the researchers observed the R482W mutation compromised PRC2 binding to the *TBXT* locus, associated with its upregulation and subsequent abnormal endothelial cell differentiation in R438W hiPSCs compared to corrected control cells. Collectively, these results link loss of LAD organization to gene expression changes with significant phenotypic consequences.

Our group recently investigated two different pathogenic *LMNA* missense mutations (*LMNA* T10I and *LMNA* R541C, hereafter T10I and R541C), previously linked to *LMNA*-associated DCM [58]. We generated independent hiPSC lines with the pathogenic mutations and isogenic controls and determined the impact of the mutations on genome organization following cardiac differentiation. We observed gross nuclear morphology defects and reduced H3K9me2 enrichment at the nuclear periphery. We also observed that the majority of LADs in isogenic control hiPSC-cardiac myocytes remained stable in mutant (~75%), but approximately 25% were lost in mutant cells. Patients with these mutations developed DCM, so we differentiated cells into two other cell types (hepatocytes and adipocytes). Perhaps surprisingly, LADs remained relatively unaffected in mutant hepatocytes and adipocytes compared to control. Notably, most LADs lost in T10I and R541C hiPSC-cardiac myocytes compared to control cells shared specific molecular features: higher gene density, reduced LB1 enrichment, and smaller in size. Further analysis of the data demonstrated that genes relating to non-cardiac myocyte fate showed a loss of lamina association and were misexpressed in the mutant hiPSC-cardiac myocytes compared to control. Re-analysis of publicly available datasets demonstrated that parallel changes were observed in *LMNA* myocardium compared to idiopathic DCM. Similarly, *LMNA* haploinsufficient cardiac myocytes also demonstrated loss of normal LAD architecture [44,57]. Furthermore, LADs were shown to be disrupted in human myocardium at the time of explant in patients harboring *LMNA* mutations [68]. Additional support for LAD dysregulation comes from an *ex vivo* study of patient myocardium and fibroblasts with *LMNA* E161K, a *LMNA* mutation associated with DCM [91]. The research team closely examined two gene clusters associated with striated muscle dysfunction and discovered repositioning of those clusters away from the nuclear periphery by DNA FISH. Additionally, they observed reduced compaction and mis-localization of chromosome 13, on which many genes were aberrantly expressed.

The upregulation of non-lineage genes is not restricted to *LMNA*-associated DCM. A group of investigators assessed the impact on LADs of two different pathogenic *LMNA* mutations linked to EDMD (*LMNA* R453W) and FPLD (*LMNA* R482W) in skeletal myoblasts [88]. The team found that *SOX2*, a pluripotency gene, lost lamina-association in both mutations, a finding consistent with EDMD patient muscle biopsies that demonstrated abnormal *SOX2* expression. Importantly, when they overexpressed SOX2 in normal myoblasts, they confirmed that this inhibited myogenic differentiation [88]. This study underscores the detrimental impact of aberrantly expressed non-lineage genes in laminopathy cells on cellular function and points to possible mechanisms through which laminopathy pathogenesis may occur. The continued use of various models and mutations will provide important datasets to reveal pathways impacted by different mutations, as well as primary versus secondary changes, such as those related to decreased cardiac contractile function, fibrosis, and age.

**Figure 1.**
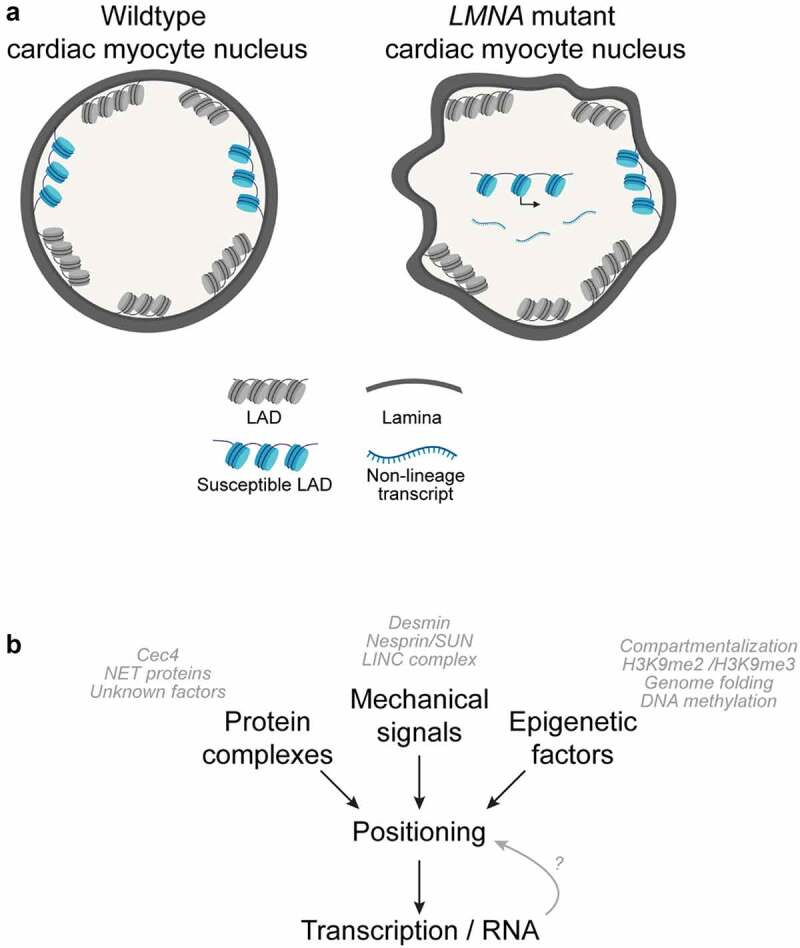
Aberrant chromatin positioning is implicated in laminopathy disease. (a) *LMNA* mutant cells exhibit defects in gross nuclear morphology and feature aberrantly localized regions of chromatin that are normally lamina-associated in cells without disease. Aberrantly expressed non-lineage genes are correlated to regions that lose lamina-association in pathogenic *LMNA* mutant cardiac myocytes. (b) Multiple molecular factors and pathways contribute to normal genome organization and integrity, including positioning factors (such as tethering proteins), mechanical force, and epigenetic factors. Laminopathies have been linked to a number of aberrant phenotypes in these pathways, and it is interesting to consider if abnormal chromatin positioning/genome organization is a shared phenotype in various pathogenic *LMNA* mutations.

Taken together, these studies support a model in which *LMNA* mutations result in a loss of cellular identity due to LAD organization defects (Figure 1). In such a model, dissociated LADs result in an ‘epigenetically vulnerable’ cell state where the misloca-lization of non-lineage genes increases the possibility of aberrant gene expression. This results in toxicity to the cell and/or inappropriate cellular plasticity, ultimately contributing to the clinical manifestations of laminopathies. Much work remains to verify tenets of this model, and the systems in which these studies are executed must be adequately considered. First, it remains necessary to test the effect of the expression of non-lineage genes more comprehensively and understand how only certain cell types manifest aberrant phenotypes in pathologic *LMNA* mutants. Advances in synthetic biology to re-localize multiple loci simultaneously [92] will be essential in these mechanistic efforts to directly test whether manipulating genome organization can rescue disease phenotypes. Second, it is possible that organization into functional tissues modifies some of the changes observed in *LMNA* mutant cells differentiated in 2D contexts, especially noting the immaturity of *in vitro* iPSC-derived cells, as discussed above. This is especially important to consider given the evidence that mechanical stress causes genomic instability in laminopathies [93]. The intersection of mechanotransduction and laminopathies is reviewed in detail elsewhere [19,94], and there is a growing appreciation for the interrelationship between mechanotransduction, genome organization, and gene expression. Moreover, advancing tissue engineering approaches will more accurately probe the role of cell-cell interactions within tissue [95]. Lastly, it must also be considered that genome organization changes may accumulate over time, especially in non-dividing cells.

Importantly, the specific temporal and cause/effect relationship between LAD positioning, condensation, and gene expression must also be more directly examined (Figure 1). For example, in the proposed model, aberrant transcription may be a consequence of LAD repositioning, resulting from a previously inaccessible gene becoming inappropriately competent for transcription once repositioned away from the repressive environment of the nuclear periphery. However, it is not known if disassociated LAD regions in pathogenic *LMNA* cells become decondensed after release from the periphery or if decondensation drives their release. Likewise, does aberrant transcription drive decondensation/disassociation or is aberrant transcription a consequence of decondensation/disassociation from the lamina? To that end, defining the determinants of LAD positioning is critical. In *C. elegans* embryos, the chromodomain protein CEC-4 has been shown to be bound to the inner nuclear membrane and to function as a tether of H3K9me-modified heterochromatin; however, no mammalian orthologue has been identified [96]. Intriguingly, loss of CEC-4 in a model of EDMD rescued LAD organization and other muscle-related phenotypes [97]. Similarly, in murine studies, three different muscle-specific nuclear envelope transmembrane proteins (NET39, Tmem38A, and WFS1) were shown to direct myogenic genes to the nuclear periphery, and a NET39 fragment targeted to the nucleolus repositioned a NET39 target gene [80]. Further studies by the Gasser lab have shown that normal spatial organization of *C. elegans* chromatin involves both perinuclear attachment of heterochromatin *and* active retention of CBP-1/p300-bound euchromatin, with the different pathways contributing differentially between embryos and larval cells [98].

Given the paucity of tethering mechanisms elucidated in mammalian models, one approach is to screen genome-wide for positioning factors. Such a screen would prioritize factors that directly or indirectly function to tether chromatin to the lamina. For example, it may identify epigenetic enzymes critical for specific lamina-associated chromatin modifications, such as H3K9me2/3, which are implicated in regulating heterochromatin formation, as discussed above. Perhaps more excitingly, an unbiased approach may reveal factors driving other potential mechanisms relating to spatial positioning, such as phase separation-mediated [99], RNA-mediated [100–102], cytoskeletal proteins, and cohesin/looping-mediated genome organization [76,103,104]. As discussed above, Hi-C assays show higher order genome organization and LAD maps reveal positioning information for regions of DNA at the lamina. Basic integration of these mapping strategies shows that LADs overwhelmingly map to the B compartment [73], which suggests that LADs may be impacted or regulated by similar factors that dictate higher order genome organization. These complementary approaches should be more closely evaluated in future studies to identify and better understand the relationship between factors that regulate genome organization and positioning. Finally, our group and others are also focused on understanding the diversity of LADs, underscoring that not all LADs are the ‘same’ [75,82,105]. In the context of RNA mediating genome organization, it is interesting to further explore how transcription at gene-rich LAD borders and/or regions of higher gene density in subsets of ‘weaker’ LADs impacts LAD organization. Relatedly, it is possible that LAD positioning, condensation, or transcriptional changes leave cells in an epigenetically vulnerable state that is exacerbated over time by normal physiological functions.

## Conclusion

Murine and hiPSC models have propelled the molecular study of laminopathies, and we expect the rate of discovery to continue to increase. As continued discovery uncovers the consequences of multiple molecular pathways on genome organization, it will be critical to understand the interplay between these pathways and the nuclear lamina in laminopathy-associated genome organization and transcriptional change. Such an understanding will greatly facilitate the development of novel therapeutics to treat laminopathies.

## References

[1] Patil S, Sengupta K. Role of A- and B-type lamins in nuclear structure-function relationships. Biol Cell. 2021 Jul;113(7):295–310. 33638183 10.1111/boc.202000160

[2] Shevelyov YY, Ulianov SV. The nuclear lamina as an organizer of chromosome architecture. Cells. 2019 Feb 8;8(2):136. 30744037 10.3390/cells8020136 PMC6406483

[3] Worman HJ, Bonne G. ”Laminopathies”: a wide spectrum of human diseases. Exp Cell Res. 2007 Jun 10;313(10):2121–2133. 17467691 10.1016/j.yexcr.2007.03.028 PMC2964355

[4] Heidenreich PA, Bozkurt B, Aguilar D, et al. 2022 AHA/ACC/HFSA guideline for the management of heart failure: a report of the American College of Cardiology/American Heart Association joint committee on clinical practice guidelines. J Am Coll Cardiol. 2022 May 3;79(17):e263–e421. 35379503 10.1016/j.jacc.2021.12.012

[5] Kang PB, Morrison L, Iannaccone ST, et al. Evidence-based guideline summary: evaluation, diagnosis, and management of congenital muscular dystrophy: report of the guideline development subcommittee of the American Academy of Neurology and the practice issues review panel of the American Association of Neuromuscular & Electrodiagnostic Medicine. Neurology. 2015 Mar 31;84(13):1369–1378. 25825463 10.1212/WNL.0000000000001416 PMC4388744

[6] Narayanaswami P, Weiss M, Selcen D, et al. Evidence-based guideline summary: diagnosis and treatment of limb-girdle and distal dystrophies: report of the guideline development subcommittee of the American Academy of Neurology and the practice issues review panel of the American Association of Neuromuscular & Electrodiagnostic Medicine. Neurology. 2014 Oct 14;83(16):1453–1463. 25313375 10.1212/WNL.0000000000000892 PMC4206155

[7] De Sandre-Giovannoli A, Chaouch M, Kozlov S, et al. Homozygous defects in LMNA, encoding lamin A/C nuclear-envelope proteins, cause autosomal recessive axonal neuropathy in human (Charcot-Marie-Tooth disorder type 2) and mouse. Am J Hum Genet. 2002 Mar;70(3):726–736. 11799477 10.1086/339274 PMC384949

[8] Gordon LB, Brown WT, Collins FS, et al. ”Hutchinson-gilford progeria syndrome,” in *GeneReviews((R))* In : Adam MP, editors. Seattle (WA): University of Washington; 1993. 20301300

[9] Burke B, Stewart CL. The nuclear lamins: flexibility in function. Nat Rev Mol Cell Biol. 2013 Jan;14(1):13–24. 23212477 10.1038/nrm3488

[10] Rankin J, Ellard S. The laminopathies: a clinical review. Clin Genet. 2006 Oct;70(4):261–274. 16965317 10.1111/j.1399-0004.2006.00677.x

[11] Wolf CM, Wang L, Alcalai R, et al. Lamin A/C haploinsufficiency causes dilated cardiomyopathy and apoptosis-triggered cardiac conduction system disease. J Mol Cell Cardiol. 2008 Feb;44(2):293–303. 18182166 10.1016/j.yjmcc.2007.11.008 PMC3011813

[12] Nikolova V, Leimena C, McMahon AC, et al. Defects in nuclear structure and function promote dilated cardiomyopathy in lamin A/C-deficient mice. J Clin Invest. 2004 Feb;113(3):357–369. 14755333 10.1172/JCI19448 PMC324538

[13] Captur G, Arbustini E, Syrris P, et al. Lamin mutation location predicts cardiac phenotype severity: combined analysis of the published literature. Open Heart. 2018;5(2):e000915. 30402260 10.1136/openhrt-2018-000915 PMC6203068

[14] Captur G, Bilinska Z, Arbustini E. Lamin missense mutations-the spectrum of phenotype variability is increasing. Eur J Heart Fail. 2018 Oct;20(10):1413–1416. 30178466 10.1002/ejhf.1290

[15] Brodsky GL, Muntoni F, Miocic S, et al. Lamin A/C gene mutation associated with dilated cardiomyopathy with variable skeletal muscle involvement. Circulation. 2000 Feb 8;101(5):473–476. 10662742 10.1161/01.cir.101.5.473

[16] Capell BC, Collins FS. Human laminopathies: nuclei gone genetically awry. Nat Rev Genet. 2006 Dec;7(12):940–952. 17139325 10.1038/nrg1906

[17] Crasto S, My I, Di Pasquale E. The broad spectrum of LMNA Cardiac diseases: from molecular mechanisms to clinical phenotype. Front Physiol. 2020;11:761. 32719615 10.3389/fphys.2020.00761 PMC7349320

[18] Briand N, Collas P. Laminopathy-causing lamin A mutations reconfigure lamina-associated domains and local spatial chromatin conformation. Nucleus. 2018 Jan 1;9(1):216–226. 29517398 10.1080/19491034.2018.1449498 PMC5973257

[19] Donnaloja F, Carnevali F, Jacchetti E, et al. Lamin A/C mechanotransduction in laminopathies. Cells. 2020 May 24;9(5):1306. 32456328 10.3390/cells9051306 PMC7291067

[20] Maraldi NM, Capanni C, Cenni V, et al. Laminopathies and lamin-associated signaling pathways. J Cell Biochem. 2011 Apr;112(4):979–992. 21400569 10.1002/jcb.22992

[21] Muchir A, Pavlidis P, Decostre V, et al. Activation of MAPK pathways links LMNA mutations to cardiomyopathy in Emery-Dreifuss muscular dystrophy. J Clin Invest. 2007 May;117(5):1282–1293. 17446932 10.1172/JCI29042 PMC1849984

[22] Muchir A, Worman HJ. Targeting mitogen-activated protein kinase signaling in mouse models of cardiomyopathy caused by lamin A/C gene mutations. Methods Enzymol. 2016;568:557–580. 26795484 10.1016/bs.mie.2015.07.028 PMC4878678

[23] Wu W, Muchir A, Shan J, et al. Mitogen-activated protein kinase inhibitors improve heart function and prevent fibrosis in cardiomyopathy caused by mutation in lamin A/C gene. Circulation. 2011 Jan 4;123(1):53–61 21173351 10.1161/CIRCULATIONAHA.110.970673 PMC3061281

[24] Brull A, Morales Rodriguez B, Bonne G, et al. The pathogenesis and therapies of striated muscle laminopathies. Front Physiol. 2018;9:1533 30425656 10.3389/fphys.2018.01533 PMC6218675

[25] Gerace L, Tapia O. Messages from the voices within: regulation of signaling by proteins of the nuclear lamina. Curr Opin Cell Biol. 2018 Jun;52:14–21 29306725 10.1016/j.ceb.2017.12.009 PMC5988997

[26] Pfizer I. Pfizer to discontinue development program for PF-07265803 for LMNA-related dilated cardiomyopathy. [cited 8 Apr 2022]. https://www.pfizer.com/news/announcements/pfizer-discontinue-development-program-pf-07265803-lmna-related-dilated.

[27] Nmezi B, Xu J, Fu R, et al. Concentric organization of A- and B-type lamins predicts their distinct roles in the spatial organization and stability of the nuclear lamina. Proc Natl Acad Sci U S A. 2019 Mar 5;116(10):4307–4315 30765529 10.1073/pnas.1810070116 PMC6410836

[28] Sullivan T, Escalante-Alcalde D, Bhatt H, et al. Loss of A-type lamin expression compromises nuclear envelope integrity leading to muscular dystrophy. J Cell Biol. 1999 Nov 29;147(5):913–920 10579712 10.1083/jcb.147.5.913 PMC2169344

[29] Swift J, Discher DE. The nuclear lamina is mechano-responsive to ECM elasticity in mature tissue. J Cell Sci. 2014 Jul 15;127(Pt 14):3005–3015 24963133 10.1242/jcs.149203 PMC4095853

[30] Davies PF, Tripathi SC. Mechanical stress mechanisms and the cell. An endothelial paradigm. Circ Res. 1993 Feb;72(2):239–245 8418981 10.1161/01.res.72.2.239

[31] Martino F, Perestrelo AR, Vinarsky V, et al. Cellular mechanotransduction: from tension to function. Front Physiol. 2018;9:824. 30026699 10.3389/fphys.2018.00824 PMC6041413

[32] Malashicheva A, Perepelina K. Diversity of nuclear lamin A/C action as a key to tissue-specific regulation of cellular identity in health and disease. Front Cell Dev Biol. 2021;9:761469. 34722546 10.3389/fcell.2021.761469 PMC8548693

[33] Avagliano L, Parenti I, Grazioli P, et al. Chromatinopathies: a focus on Cornelia de Lange syndrome. Clin Genet. 2020 Jan;97(1):3–11 31721174 10.1111/cge.13674

[34] Barutcu AR, Lajoie BR, McCord RP, et al. Chromatin interaction analysis reveals changes in small chromosome and telomere clustering between epithelial and breast cancer cells. Genome Biol. 2015 Sep 28;16(1):214 26415882 10.1186/s13059-015-0768-0 PMC4587679

[35] De Gobbi M, Viprakasit V, Hughes JR, et al. A regulatory SNP causes a human genetic disease by creating a new transcriptional promoter. Science. 2006 May 26;312(5777):1215–1217 16728641 10.1126/science.1126431

[36] Lupianez DG, Kraft K, Heinrich V, et al. Disruptions of topological chromatin domains cause pathogenic rewiring of gene-enhancer interactions. Cell. 2015 May 21;161(5):1012–1025 25959774 10.1016/j.cell.2015.04.004 PMC4791538

[37] Dixon JR, Selvaraj S, Yue F, et al. Topological domains in mammalian genomes identified by analysis of chromatin interactions. Nature. 2012 Apr 11;485(7398):376–380 22495300 10.1038/nature11082 PMC3356448

[38] Tolhuis B, Palstra RJ, Splinter E, et al. Looping and interaction between hypersensitive sites in the active beta-globin locus. Mol Cell. 2002 Dec;10(6):1453–1465 12504019 10.1016/s1097-2765(02)00781-5

[39] Hsieh TS, Cattoglio C, Slobodyanyuk E, et al. Resolving the 3D landscape of transcription-linked mammalian chromatin folding. Mol Cell. 2020 May 7;78(3):539–553 e8 32213323 10.1016/j.molcel.2020.03.002 PMC7703524

[40] Belton JM, McCord RP, Gibcus JH, et al. Hi-C: a comprehensive technique to capture the conformation of genomes. Methods. 2012 Nov;58(3):268–276 22652625 10.1016/j.ymeth.2012.05.001 PMC3874846

[41] Lieberman-Aiden E, van Berkum NL, Williams L, et al. Comprehensive mapping of long-range interactions reveals folding principles of the human genome. Science. 2009 Oct 9;326(5950):289–293 19815776 10.1126/science.1181369 PMC2858594

[42] Krietenstein N, Abraham S, Venev SV, et al. Ultrastructural details of mammalian chromosome architecture. Mol Cell. 2020 May 7;78(3):554–565 e7 32213324 10.1016/j.molcel.2020.03.003 PMC7222625

[43] Zheng X, Hu J, Yue S, et al. Lamins organize the global three-dimensional genome from the nuclear periphery. Mol Cell. 2018 Sep 6;71(5):802–815 e7 30201095 10.1016/j.molcel.2018.05.017 PMC6886264

[44] Bertero A, Fields PA, Smith AST, et al. Chromatin compartment dynamics in a haploinsufficient model of cardiac laminopathy. J Cell Biol. 2019 Sep 2;218(9):2919–2944 31395619 10.1083/jcb.201902117 PMC6719452

[45] Mozzetta C, Tedesco FS. Challenging the ”chromatin hypothesis” of cardiac laminopathies with LMNA mutant iPS cells. J Cell Biol. 2019 Sep 2;218(9):2826–2828 31427369 10.1083/jcb.201907166 PMC6719444

[46] van Berkum NL, Lieberman-Aiden E, Williams L, et al. Hi-C: a method to study the three-dimensional architecture of genomes. J Vis Exp. 2010 May 6;(39): DOI: 10.3791/1869. PMC3149993 20461051

[47] Bintu B, Mateo LJ, Su J-H, et al. Super-resolution chromatin tracing reveals domains and cooperative interactions in single cells. Science. 2018 Oct 26;362(6413): 10.1126/science.aau1783. PMC6535145 30361340

[48] Mateo LJ, Murphy SE, Hafner A, et al. Visualizing DNA folding and RNA in embryos at single-cell resolution. Nature. 2019 Apr;568(7750):49–54 30886393 10.1038/s41586-019-1035-4 PMC6556380

[49] Neguembor MV, Martin L, Castells-García Á, et al. Transcription-mediated supercoiling regulates genome folding and loop formation. Mol Cell. 2021 Aug 5;81(15):3065–3081 e12 34297911 10.1016/j.molcel.2021.06.009 PMC9482096

[50] Ivashchenko CY, Pipes GC, Lozinskaya IM, et al. Human-induced pluripotent stem cell-derived cardiomyocytes exhibit temporal changes in phenotype. Am J Physiol Heart Circ Physiol. 2013 Sep 15;305(6):H913–22 23832699 10.1152/ajpheart.00819.2012

[51] Margueron R, Reinberg D. The polycomb complex PRC2 and its mark in life. Nature. 2011 Jan 20;469(7330):343–349 21248841 10.1038/nature09784 PMC3760771

[52] Petracovici A, Bonasio R. Distinct PRC2 subunits regulate maintenance and establishment of Polycomb repression during differentiation. Mol Cell. 2021 Jun 17;81(12):2625–2639 e5 33887196 10.1016/j.molcel.2021.03.038 PMC8217195

[53] Cesarini E, Mozzetta C, Marullo F, et al. Lamin A/C sustains PcG protein architecture, maintaining transcriptional repression at target genes. J Cell Biol. 2015 Nov 9;211(3):533–551 26553927 10.1083/jcb.201504035 PMC4639869

[54] Salvarani N, Crasto S, Miragoli M, et al. The K219T-Lamin mutation induces conduction defects through epigenetic inhibition of SCN5A in human cardiac laminopathy. Nat Commun. 2019 May 22;10(1):2267 31118417 10.1038/s41467-019-09929-w PMC6531493

[55] Wiehle L, Breiling A. Chromatin Immunoprecipitation. Methods Mol Biol. 2016;1480:7–21 27659971 10.1007/978-1-4939-6380-5_2

[56] Bayani J, Squire JA. Fluorescence in situ hybridization (FISH. Curr Protoc Cell Biol. 2004 Sep;22:22 4 10.1002/0471143030.cb2204s23 18228455

[57] Lee J, Termglinchan V, Diecke S, et al. Activation of PDGF pathway links LMNA mutation to dilated cardiomyopathy. Nature. 2019 Aug;572(7769):335–340 31316208 10.1038/s41586-019-1406-x PMC6779479

[58] Shah PP, Lv W, Rhoades JH, et al. Pathogenic LMNA variants disrupt cardiac lamina-chromatin interactions and de-repress alternative fate genes. Cell Stem Cell. 2021 May 6;28(5):938–954 e9 33529599 10.1016/j.stem.2020.12.016 PMC8106635

[59] Oldenburg A, Briand N, Sørensen AL, et al. A lipodystrophy-causing lamin A mutant alters confor-mation and epigenetic regulation of the anti-adipogenic MIR335 locus. J Cell Biol. 2017 Sep 4;216(9):2731–2743 28751304 10.1083/jcb.201701043 PMC5584164

[60] Poleshko A, Shah PP, Gupta M, et al. Genome-nuclear lamina interactions regulate cardiac stem cell lineage restriction. Cell. 2017 Oct 19;171(3):573–587 e14 29033129 10.1016/j.cell.2017.09.018 PMC5683101

[61] Poleshko A, et al. H3K9me2 orchestrates inheritance of spatial positioning of peripheral heterochromatin through mitosis. Elife. 2019 Oct 1;8(9):2826–2828 10.7554/eLife.49278 PMC6795522 31573510

[62] Zheng M, Jin G, Zhou Z. Post-translational modification of lamins: mechanisms and functions. Front Cell Dev Biol. 2022;10:864191. 35656549 10.3389/fcell.2022.864191 PMC9152177

[63] Marullo F, Cesarini E, Antonelli L, et al. Nucleoplasmic lamin A/C and polycomb group of proteins: an evolutionarily conserved interplay. Nucleus. 2016 Apr 25;7(2):103–111 26930442 10.1080/19491034.2016.1157675 PMC4916880

[64] Kochin V, Shimi T, Torvaldson E, et al. Interphase phosphorylation of lamin A. J Cell Sci. 2014 Jun 15;127(Pt 12):2683–2696. 24741066 10.1242/jcs.141820 PMC4058112

[65] Liu SY, Ikegami K. Nuclear lamin phosphorylation: an emerging role in gene regulation and pathogenesis of laminopathies. Nucleus. 2020 Dec;11(1):299–314 33030403 10.1080/19491034.2020.1832734 PMC7588210

[66] Ikegami K, Secchia S, Almakki O, et al. Phosphorylated lamin A/C in the nuclear interior binds active enhancers associated with abnormal transcription in progeria. Dev Cell. 2020 Mar 23;52(6):699–713 e11 32208162 10.1016/j.devcel.2020.02.011 PMC7201903

[67] Morival JLP, Widyastuti HP, Nguyen CHH, et al. DNA methylation analysis reveals epimutation hotspots in patients with dilated cardiomyopathy-associated laminopathies. Clin Epigenetics. 2021 Jul 10;13(1):139 34246298 10.1186/s13148-021-01127-0 PMC8272901

[68] Cheedipudi SM, Matkovich SJ, Coarfa C, et al. Genomic reorganization of lamin-associated domains in cardiac myocytes is associated with differential gene expression and DNA methylation in human dilated cardiomyopathy. Circ Res. 2019 Apr 12;124(8):1198–1213 30739589 10.1161/CIRCRESAHA.118.314177 PMC6459729

[69] Watanabe T, Okada H, Kanamori H, et al. ”In situ nuclear DNA methylation in dilated cardiomyopathy: an endomyocardial biopsy study,”. ESC Heart Fail. 2020 Apr;7(2):493–502 31971668 10.1002/ehf2.12593 PMC7160509

[70] Chen X, Yammine S, Shi C, et al. The visualization of large organized chromatin domains enriched in the H3K9me2 mark within a single chromosome in a single cell. Epigenetics. 2014 Nov;9(11):1439–1445. 25482057 10.4161/15592294.2014.971633 PMC4623470

[71] Guelen L, Pagie L, Brasset E, et al. Domain organization of human chromosomes revealed by mapping of nuclear lamina interactions. Nature. 2008 Jun 12;453(7197):948–951 18463634 10.1038/nature06947

[72] Wen B, Wu H, Shinkai Y, et al. Large histone H3 lysine 9 dimethylated chromatin blocks distinguish differentiated from embryonic stem cells. Nat Genet. 2009 Feb;41(2):246–250 19151716 10.1038/ng.297 PMC2632725

[73] Luperchio T, Sauria MEG, Hoskins VE, et al. The repressive genome compartment is established early in the cell cycle before forming the lamina associated domains. bioRxiv. 2018;481598. DOI:10.1101/481598.

[74] Kind J, van Steensel B. Genome-nuclear lamina interactions and gene regulation. Curr Opin Cell Biol. 2010 Jun;22(3):320–325. 20444586 10.1016/j.ceb.2010.04.002

[75] Keough KC, Shah PP, Gjoni K, et al. An atlas of lamina-associated chromatin across twelve human cell types reveals an intermediate chromatin subtype. bioRxiv. 2020; DOI:10.1101/2020.07.23.218768. Art no. 2020.07.23.218768. PMC9869549 36691074

[76] van Schaik T, Liu NQ, Manzo SG, et al. CTCF and cohesin promote focal detachment of DNA from the nuclear lamina. bioRxiv. 2021;Art no. 2021.09.13.460079. doi:10.1101/2021.09.13.460079 PMC9438259 36050765

[77] Peric-Hupkes D, Meuleman W, Pagie L, et al. Molecular maps of the reorganization of genome-nuclear lamina interactions during differentiation. Mol Cell. 2010 May 28;38(4):603–613 20513434 10.1016/j.molcel.2010.03.016 PMC5975946

[78] Zullo JM, Demarco I, Piqué-Regi R, et al. DNA sequence-dependent compartmentalization and silencing of chromatin at the nuclear lamina. Cell. 2012 Jun 22;149(7):1474–1487. 22726435 10.1016/j.cell.2012.04.035

[79] Meister P, Towbin BD, Pike BL, et al. The spatial dynamics of tissue-specific promoters during C. elegans development. Genes Dev. 2010 Apr 15;24(8):766–782 20395364 10.1101/gad.559610 PMC2854392

[80] Robson MI, De las heras J, Czapiewski R, et al. Tissue-specific gene repositioning by muscle nuclear membrane proteins enhances repression of critical developmental genes during myogenesis. Mol Cell. 2016 Jun 16;62(6):834–847 27264872 10.1016/j.molcel.2016.04.035 PMC4914829

[81] Smith CL, Lan Y, Jain R, et al. Global chromatin relabeling accompanies spatial inversion of chromatin in rod photoreceptors. Sci Adv. 2021 Sep 24;7(39):eabj3035. 34559565 10.1126/sciadv.abj3035 PMC8462898

[82] Meuleman W, Peric-Hupkes D, Kind J, et al. Constitutive nuclear lamina-genome interactions are highly conserved and associated with A/T-rich sequence. Genome Res. 2013 Feb;23(2):270–280. 23124521 10.1101/gr.141028.112 PMC3561868

[83] Cavaliere V, Lattanzi G, Andrenacci D. Silencing of euchromatic transposable elements as a consequence of nuclear lamina dysfunction. Cells. 2020 Mar 5;9(3):625. 32151001 10.3390/cells9030625 PMC7140440

[84] Della Valle F, Reddy P, Yamamoto M, et al. ”LINE-1 RNA causes heterochromatin erosion and is a target for amelioration of senescent phenotypes in progeroid syndromes,”. Sci Transl Med. 2022 Aug 10;14(657):eabl6057 35947677 10.1126/scitranslmed.abl6057

[85] Briand N, Collas P. Lamina-associated domains: peripheral matters and internal affairs. Genome Biol. 2020 Apr 2;21(1):85 32241294 10.1186/s13059-020-02003-5 PMC7114793

[86] van Steensel B, Belmont AS. Lamina-associated domains: links with chromosome architecture, heterochromatin, and gene repression. Cell. 2017 May 18;169(5):780–791 28525751 10.1016/j.cell.2017.04.022 PMC5532494

[87] Luperchio TR, Wong X, Reddy KL. Genome regulation at the peripheral zone: lamina associated domains in development and disease. Curr Opin Genet Dev. 2014 Apr;25:50–61 24556270 10.1016/j.gde.2013.11.021

[88] Perovanovic J, Dell’Orso S, Gnochi VF, et al. Laminopathies disrupt epigenomic developmental programs and cell fate. Sci Transl Med. 2016 Apr 20;8(335):335ra58 10.1126/scitranslmed.aad4991 PMC4939618 27099177

[89] Briand N, Guénantin A-C, Jeziorowska D, et al. The lipodystrophic hotspot lamin A p.R482W mutation deregulates the mesodermal inducer T/Brachyury and early vascular differentiation gene networks. Hum Mol Genet. 2018 Apr 15;27(8):1447–1459 29438482 10.1093/hmg/ddy055

[90] Paulsen J, Sekelja M, Oldenburg AR, et al. Chrom3D: three-dimensional genome modeling from Hi-C and nuclear lamin-genome contacts. Genome Biol. 2017 Jan 30;18(1):21 28137286 10.1186/s13059-016-1146-2 PMC5278575

[91] Mewborn SK, Puckelwartz MJ, Abuisneineh F, et al. Altered chromosomal positioning, compaction, and gene expression with a lamin A/C gene mutation. PLoS One. 2010 Dec 14;5(12):e14342 21179469 10.1371/journal.pone.0014342 PMC3001866

[92] Wang H, Xu X, Nguyen CM, et al. CRISPR-mediated programmable 3D genome positioning and nuclear organization. Cell. 2018 Nov 15;175(5):1405–1417 e14. 30318144 10.1016/j.cell.2018.09.013 PMC6239909

[93] Lammerding J, Schulze PC, Takahashi T, et al. Lamin A/C deficiency causes defective nuclear mechanics and mechanotransduction. J Clin Invest. 2004 Feb;113(3):370–378 14755334 10.1172/JCI19670 PMC324542

[94] Osmanagic-Myers S, Dechat T, Foisner R. Lamins at the crossroads of mechanosignaling. Genes Dev. 2015 Feb 1;29(3):225–237 25644599 10.1101/gad.255968.114 PMC4318140

[95] Miura K, Matsuura K, Yamasaki Itoyama Y, et al. Functional evaluation of human bioengineered cardiac tissue using iPS cells derived from a patient with lamin variant dilated cardiomyopathy. Int Heart J. 2022;63(2):338–346. 35354754 10.1536/ihj.21-790

[96] Gonzalez-Sandoval A, Towbin B, Kalck V, et al. Perinuclear anchoring of H3K9-methylated chromatin stabilizes induced cell fate in C. elegans embryos. Cell. 2015 Dec 3;163(6):1333–1347 26607792 10.1016/j.cell.2015.10.066

[97] Harr JC, Schmid CD, Muñoz-Jiménez C, et al. Loss of an H3K9me anchor rescues laminopathy-linked changes in nuclear organization and muscle function in an emery-dreifuss muscular dystrophy model. Genes Dev. 2020 Apr 1;34(7–8):560–579 32139421 10.1101/gad.332213.119 PMC7111258

[98] Cabianca DS, Muñoz-Jiménez C, Kalck V, et al. Active chromatin marks drive spatial sequestration of heterochromatin in C. elegans nuclei. Nature. 2019 May;569(7758):734–739 31118512 10.1038/s41586-019-1243-y

[99] Keenen MM, Brown D, Brennan LD, et al. HP1 proteins compact DNA into mechanically and positionally stable phase separated domains. Elife. 2021 Mar 4;10. 10.7554/eLife.64563. PMC7932698 33661100

[100] Oh HJ, Aguilar R, Kesner B, et al. Jpx RNA regulates CTCF anchor site selection and formation of chromosome loops. Cell. 2021 Dec 9;184(25):6157–6173 e24 34856126 10.1016/j.cell.2021.11.012 PMC8671370

[101] Quinodoz SA, Jachowicz JW, Bhat P, et al. RNA promotes the formation of spatial compartments in the nucleus. Cell. 2021 Nov 11;184(23):5775–5790 e30 34739832 10.1016/j.cell.2021.10.014 PMC9115877

[102] Saldana-Meyer R, Rodriguez-Hernaez J, Escobar T, et al. RNA interactions are essential for CTCF-mediated genome organization. Mol Cell. 2019 Nov 7;76(3):412–422 e5 31522988 10.1016/j.molcel.2019.08.015 PMC7195841

[103] Haarhuis JHI, van der Weide RH, Blomen VA, et al. A mediator-cohesin axis controls heterochromatin domain formation. Nat Commun. 2022 Feb 8;13(1):754 35136067 10.1038/s41467-022-28377-7 PMC8826356

[104] Linares-Saldana R, Kim W, Bolar NA, et al. BRD4 orchestrates genome folding to promote neural crest differentiation. Nat Genet. 2021 Oct;53(10):1480–1492 34611363 10.1038/s41588-021-00934-8 PMC8500624

[105] Zheng X, Kim Y, Zheng Y. Identification of lamin B-regulated chromatin regions based on chromatin landscapes. Mol Biol Cell. 2015 Jul 15;26(14):2685–2697 25995381 10.1091/mbc.E15-04-0210 PMC4501365

